# Supplementary material for: Structural Insights into Salinosporamide a Mediated Inhibition of the Human 20S Proteasome
Source: Molecules. 2025 Mar 20;30(6):1386. doi: 10.3390/molecules30061386 (PMC11946101; doi:10.3390/molecules30061386)
Supplement: Supplementary file 1 [file molecules-30-01386-s001.zip › molecules-3496386-supplementary.pdf]

Supplementary Materials:

Structural Insights into Salinosporamide a Mediated Inhibition of the Human 20S Proteasome

Authors:

Hagen Sülzen [1], Pavla Fajtova [1,2], Anthony J. O’Donoghue [2,3], Jan Silhan [1,*] and Evzen Boura [1,*]

Affiliations:

1 Institute of Organic Chemistry and Biochemistry, Czech Academy of Sciences, Flemingovo namesti 2, 16610 Prague, Czech Republic; fajtova@uochb.cas.cz (P.F.)

2 Skaggs School of Pharmacy and Pharmaceutical Sciences, University of California San Diego, La Jolla, CA 92093, USA; ajodonoghue@health.ucsd.edu

3 Center for Discovery and Innovation in Parasitic Diseases, Skaggs School of Pharmacy and Pharmaceutical Sciences, University of California San Diego, La Jolla, CA 92093, USA

* Correspondence: jan.silhan@uochb.cas.cz (J.S.); evzen.boura@uochb.cas.cz (E.B.)

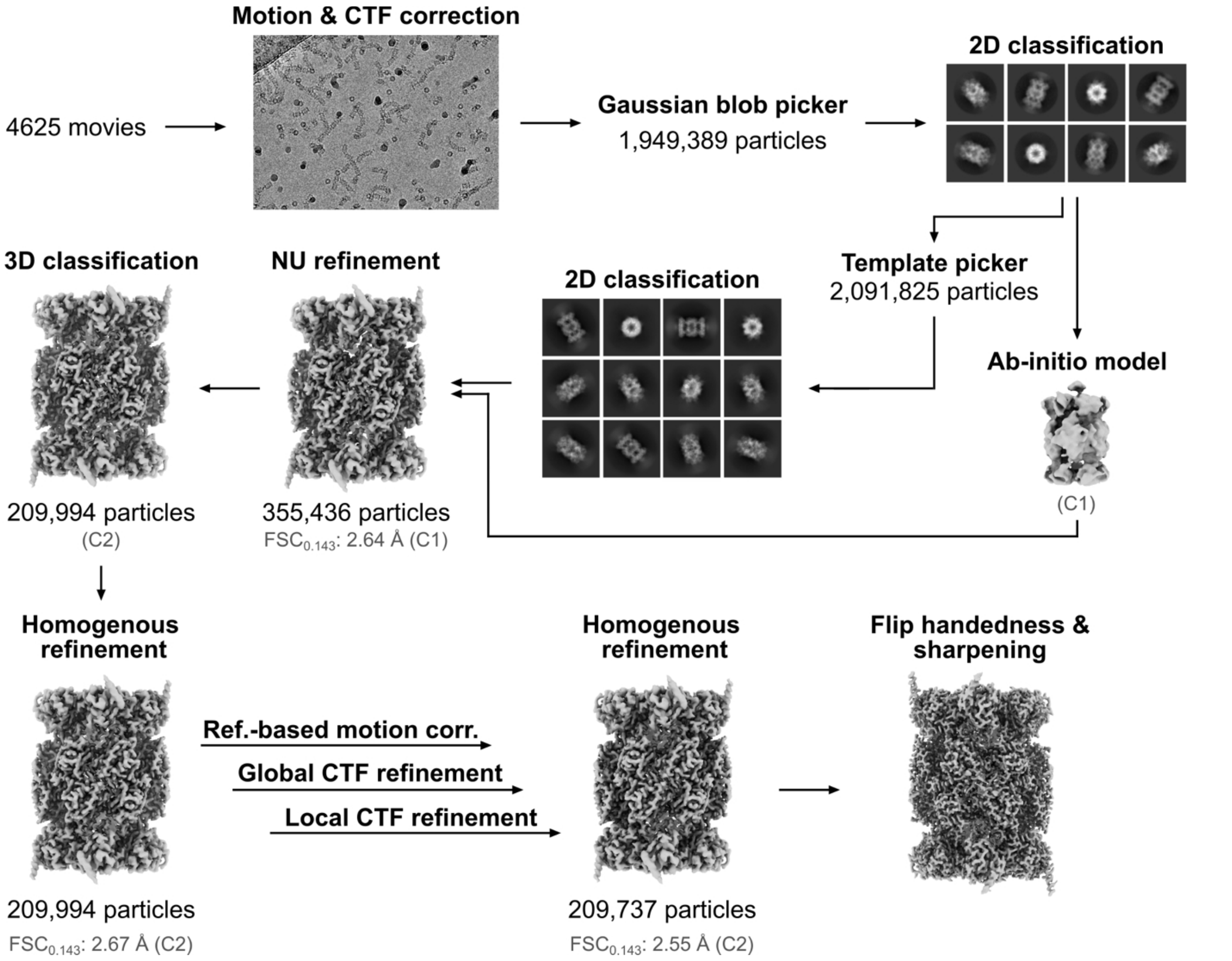

Supplementary Figure S1. Workflow of cryo-EM data processing. Visual representation of the image processing workflow used to reconstruct the h20S proteasome in complex with marizomib. A representative micrograph and selected 2D classes are shown in addition to the ab-initio model used and the density maps that were obtained for each reconstruction step. Where applicable, the number of particles, the gold-standard FSC estimate of the reconstruction resolution and the symmetry applied in the processing step (shown in brackets) are indicated. A total of 209,737 particles were used for the final reconstruction. All image processing steps were performed using cryoSPARC.[1] The final sharpening step was performed using EMReady.[2]

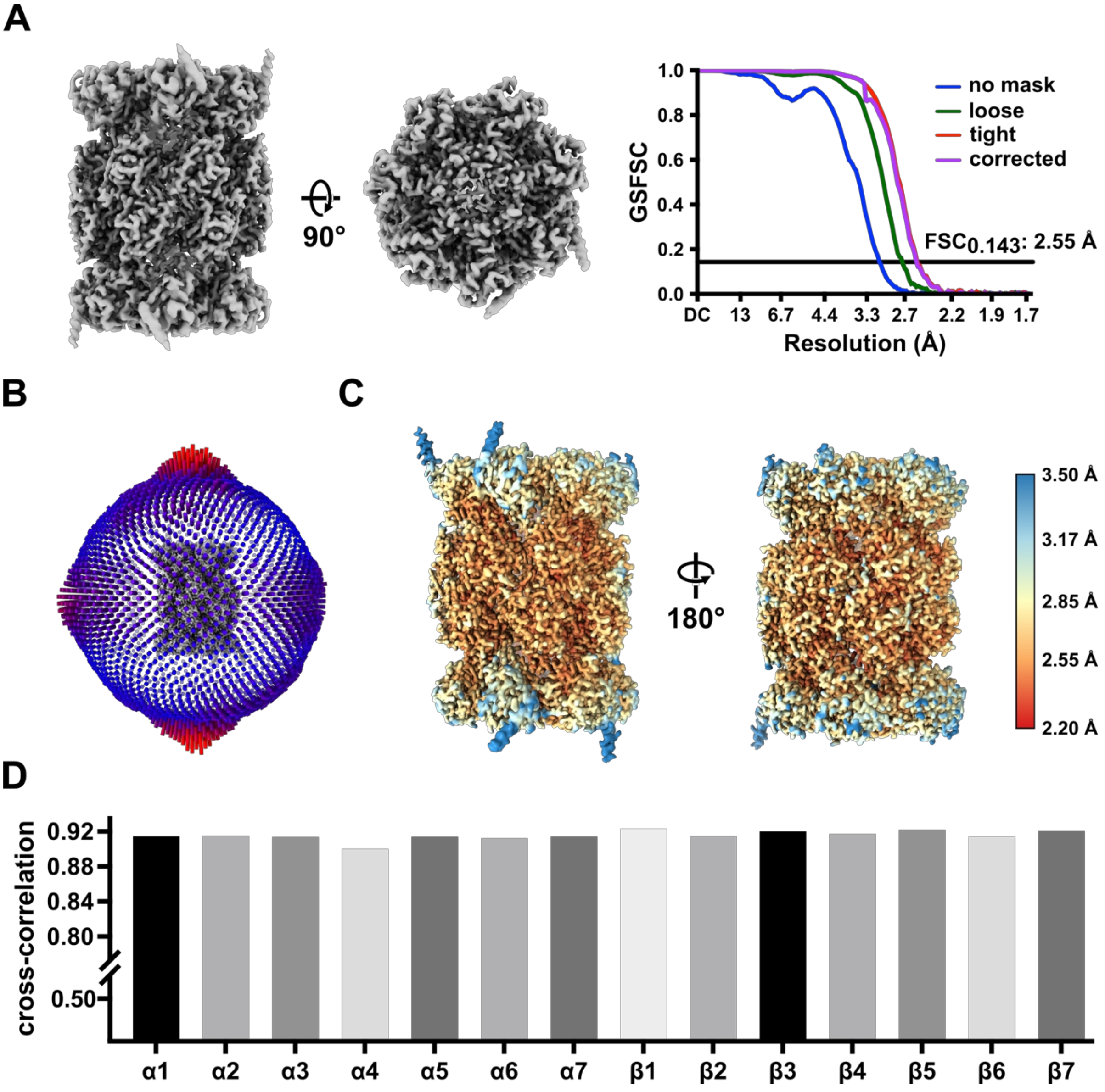

Supplementary Figure S2. Cryo-EM single particle analysis of h20S in complex with MZB. (A) Final (unsharpened) cryo-EM reconstruction (left) used to calculate the gold-standard Fourier-shell correlation curves (right). **(B)** Angular distribution plot of the final reconstruction. The height of the bars corresponds to the relative abundance of particle views contributing to the final reconstruction. The apparent symmetric distribution of Euler angles is the result of enforcing C2 symmetry during the reconstruction. **(C)** Colour-graded representation of the local resolution (in Å) of the final reconstruction, visualised on the EMReady sharpened map with corrected handedness. The range of the colour gradient was chosen to achieve the best visual representation and does not cover all spatial frequencies present in the reconstruction. **(D)** Average map-to-model cross-correlation ($CC_{side\ chain}$) for h20S subunits. Since the atomic models plotted here have been fully refined prior to symmetry expansion, the symmetry-related copies are not included here.

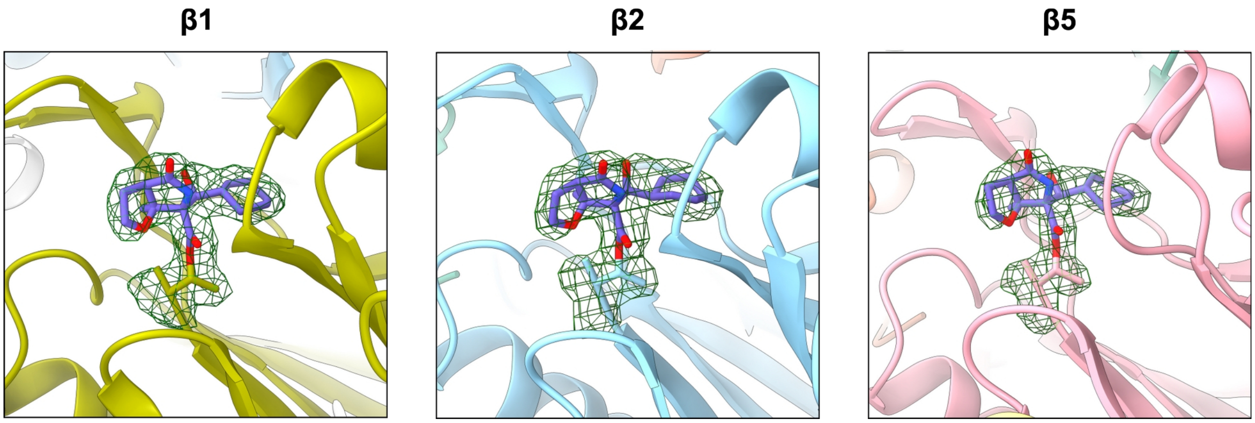

Supplementary Figure S3. Detailed views of the cryo-EM maps in active sites of human 20S with covalently bound MZB inhibitor. Sharpened cryo-EM density maps (green mesh) with highlighted MZB inhibitor (blue sticks). The β1, β2, and β5 subunits are represented as ribbons in yellow, blue, and pink, respectively.

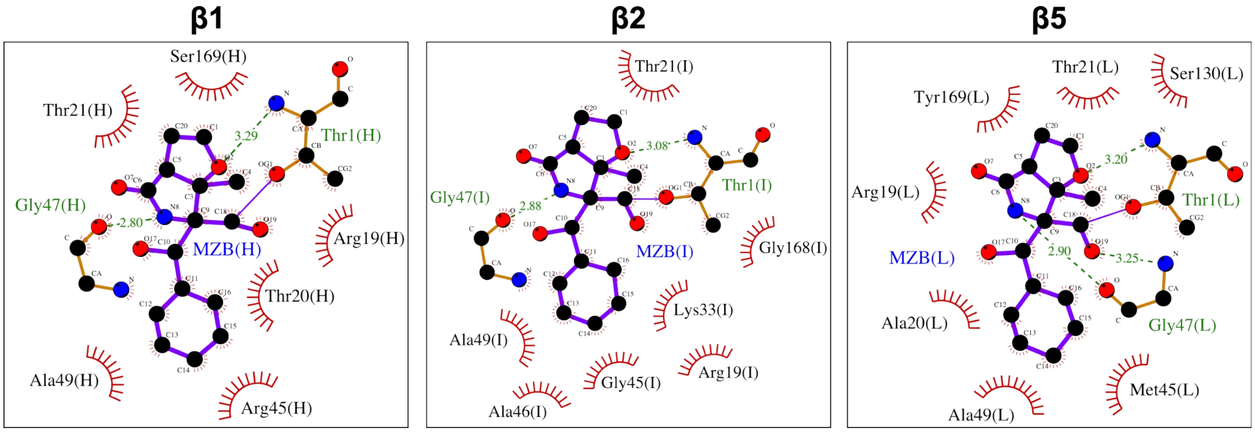

Supplementary Figure S4. 2D representation of the interactions between small molecule inhibitor MZB and the h20S active sites. Hydrogen bonds are displayed as green dashed lines, non-ligand residues involved in hydrophobic contacts are displayed as red semicircular arcs. Plots were generated using LigPlot.[3]

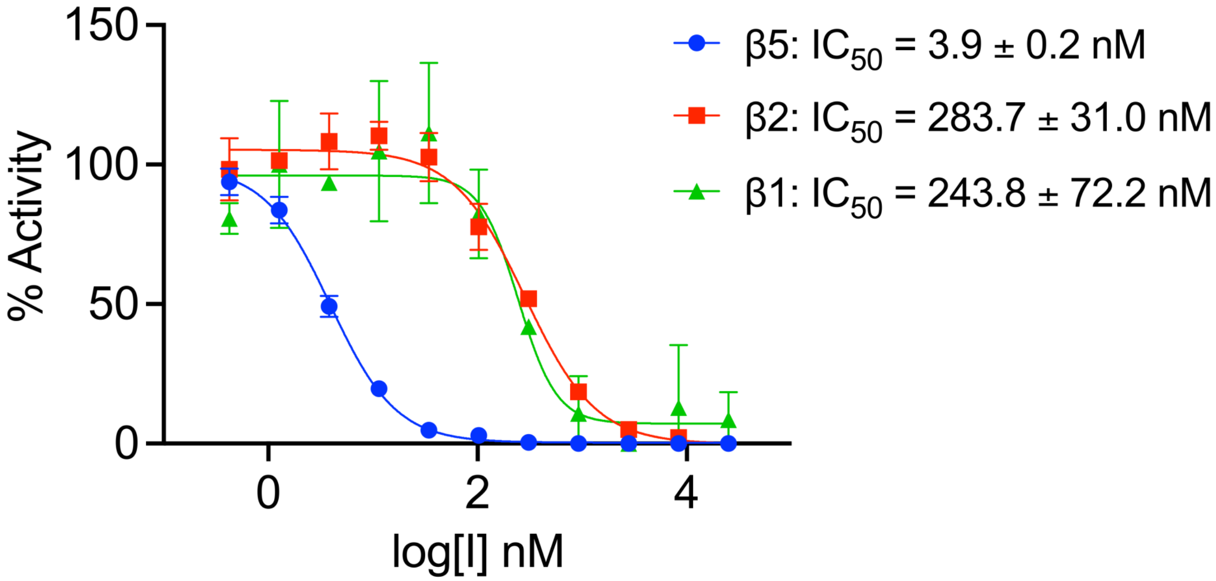

Supplementary Figure S5. Half-maximal inhibitory concentration (IC_{50}) of MZB for the proteolytic *Tv*20S subunits. IC_{50} curves for the inhibition of individual Tv20S proteasome subunits (β1, β2, and β5) determined using fluorogenic substrates Ac-RYFD-amc, Ac-FRSR-amc, and Ac-GWYL-amc. Assays were performed in 3 technical replicates in identical setup as for h20S and data are presented as mean values ± SD.

Supplementary Table S1: Cryo-EM data collection, refinement and validation statistics.

	h20S-MZB (EMDB-52296) (PDB- 9HMN)
**Data collection and processing**	
Microscope	Titan Krios
Detector	Gatan K3
Magnification (nominal)	105.000x
Voltage (kV)	300
Spherical aberration	2.7 mm
Total electron dose (e–/Å^2)	60
Defocus range (µm)	-2.5 to -1.0
Pixel size (Å)	0.8336
Stage tilt	0°
Number of Micrographs	4625
Final particle images (no.)	209,737
Map resolution (Å) [FSC threshold]	2.55 [$FSC_{0.143}$]
**Refinement**	
Initial model used (PDB code)	7PG9
Symmetry during reconstruction	C2
RMSD	
Bond lengths (Å)	0.007
Bond angles (°)	1.082
Validation	
MolProbity score	1.27
Clashscore, all-atom	5.1
Rotamer outliers	0.88%
Ramachandran plot	
Favoured	98.05%
Allowed	1.76%
Outliers	0.19%

**Model vs. Data**	
Ligands (no.)	6 (MZB)
CC (mask/box/ligand)	0.91/ 0.94 / 0.93
Resolution estimates (Å)<br>FSC (0.143/0.5)	2.55 / 2.8

Supplementary references

1 Punjani, A., Rubinstein, J. L., Fleet, D. J. & Brubaker, M. A. cryoSPARC: algorithms for rapid unsupervised cryo-EM structure determination. *Nature methods* **14**, 290-296 (2017). https://doi.org:10.1038/nmeth.4169

2 He, J., Li, T. & Huang, S. Y. Improvement of cryo-EM maps by simultaneous local and non-local deep learning. *Nature communications* **14**, 3217 (2023). https://doi.org:10.1038/s41467-023-39031-1

3 Laskowski, R. A. & Swindells, M. B. LigPlot+: multiple ligand-protein interaction diagrams for drug discovery. *Journal of chemical information and modeling* **51**, 2778-2786 (2011). https://doi.org:10.1021/ci200227u
